# Transferability of bioprocessing modes for recombinant protease production: from fed-batch to continuous cultivation with *Bacillus licheniformis*

**DOI:** 10.1186/s12896-025-00947-9

**Published:** 2025-01-31

**Authors:** Stefan Kittler, Fabian Müller, Mohamed Elshazly, Georg Benjamin Wandrey, Tobias Klein, Andreas Daub, Oliver Spadiut, Julian Kopp

**Affiliations:** 1 Research Division Integrated Bioprocess Development, Institute of Chemical, Environmental and Bioscience Engineering, TU Wien, Gumpendorfer Strasse 1a, Vienna, 1060 Austria; 2 White Biotechnology Research, BASF SE, 67063 Ludwigshafen am Rhein, Germany

**Keywords:** *Bacillus licheniformis*, Continuous processing, Model-based development, Sustainable processing, Fed-batch, Chemostat

## Abstract

**Background:**

Proteases are essential in various industries due to their unique substrate specificities and robustness in different operational conditions. *Bacillus* strains consist of a genotype favorable for rapid growth whilst secreting enzymes extracellularly, thereby simplifying recombinant protease production. Despite the widespread use of batch and fed-batch fermentations for their ease and robustness, these cultivation types are often marred by significant energy requirements and prolonged downtimes. The switch towards continuous cultivation methods promises reduced carbon footprints and improved equipment efficiency. Yet, research focusing on *Bacillus* strains is limited, therefore we aimed to establish a continuous cultivation as a competitive alternative to fed-batch.

**Results:**

Therefore, this study aimed to explore the potential of chemostat cultivations for producing a protease from *Bacillus licheniformis* utilizing a derepressed induction system, and comparing specific productivities and space-time yields to fed-batch cultivations. The continuous cultivations were described in a hybrid model, considering the effect of productivity as function of the applied dilution rate as well as the generation time. The workflow of this study demonstrates that screenings in a fed-batch mode and chemostat cultivations conducted at the same growth rate, result in different specific productivities for derepressible systems.

**Conclusion:**

The results of this study highlight that the feeding rate’s impact on specific productivity varies significantly between fed-batch and chemostat cultivations. These differences suggest that fed-batch screenings may not be adequate for developing a continuous process using a derepressed promoter system in *B. licheniformis*. Although the space-time yield of fed-batch cultivations has not been surpassed by stable continuous operations—achieving only a third of the highest space-time yield observed in fed-batch—valuable mechanistic insights have been gained. This knowledge could facilitate the transition towards a more sustainable mode of cultivation for industrial protease production.

## Background

Proteases are vital to numerous industrial applications, owing to their enzymatic activity enabling the hydrolysis of peptide bonds. As multifaceted biocatalysts, proteases have become indispensable across various sectors for their diverse and targeted applications. These enzymes are not only key ingredients in laundry detergents, enhancing the cleaning of fabrics, but they also play a significant role in pharmaceuticals, aiding in both drug formulation and disease mitigation strategies [1, 2]. In the food sector, proteases are instrumental in tenderizing meats, thus improving texture and digestibility [2]. Furthermore, their ability to rapidly decompose organic proteins makes them valuable in waste management, accelerating the degradation of organic waste [2–5]. The versatility of proteases is complemented by their substrate specificity and the conditions under which they function.

*Bacillus*, a genus of gram-positive, rod-shaped bacteria, has emerged as a host for the industrial production of proteases [6, 7]. Even though known as a spore-forming bacteria, strains employed in the food and pharmaceutical industry are genetically engineered to avoid spore formation [8]. Species like *Bacillus subtilis* and *Bacillus licheniformis* are notably known for their rapid growth rates and their ability to secrete large quantities of enzymes directly into the supernatant [9–13]. This feature simplifies the purification process, compared to many other bacterial production processes producing target molecules intracellularly. Recombinant protein production in *Bacillus licheniformis* is reported to utilize either strong constitutive, temperature-assisted, nutrient-dependent or pH-sensitive promotors [14]. Due to the high number of generations from cryo stock to production scale in industrial settings, inducible promotors are favoured, as the impact of the metabolic burden of high level protein production can be better controlled and the formation of non-producing subpopulations can be minimized [15]. Whilst pH and nutrient adjustments can be exercised quickly in an industrial production scale, cooling or heating processes can take up severe amounts of energy and time. pH changes to induce recombinant protein formation might not have a severe effect on cell growth (when maintaining physiological boundaries), however some enzymes might be prone to aggregation at given pH values. Hence, induction of recombinant protein formation in *B. licheniformis* is commonly exercised with nutrient dependent promotors in an industrial scale [16]. Promotor activity induced via phosphate limitation are well-known for *B. licheniformis*, still reduced levels of phosphate might again affect cell physiology [17]. Therefore, derepressible promotors present an elegant version, allowing recombinant enzyme production via a decreased supply of carbon source, whilst maintaining cell physiology intact [18].

Conventional cultivation modes for producing these enzymes—batch and fed-batch fermentation—are well-established in the biotechnological industry [19, 20]. Batch fermentation is straightforward: the microorganism is grown in a fixed volume of culture medium until nutrients become depleted or waste products accumulate to inhibitory levels. Hence, fed-batch cultivation is commonly exercised in microbial recombinant protein production processes. Compared to batch cultivations, substrate inhibition, osmotic stress, as well as overflow metabolism can be circumvented, facilitating elongated production conditions and higher product yields. However, time-dependent product quality and quantity is still a major drawback in fed-batches [21]. Furthermore, fed-batch processing is associated with large fermentation vessels causing for a high energy demand and long downtimes due to sterilization and cleaning, reducing the production time [22]. Therefore, economical and budget-friendly processing techniques are of growing interest and are on the rise to compete with this established cultivation strategy [23, 24].

In chemostat cultivations, a continuous substrate feed is maintained, concomitantly with continuous harvest of cell broth, allowing for a sustained equilibrium in production conditions [25, 26]. Targeted growth rates are adjusted by the ratio between the volumetric flow rate of the medium entering the reactor and the cultivation volume, denoted as the dilution rate (D). Due to long-lasting production conditions (compared to fed-batch cultivation), equipment utilization is enhanced, allowing for elevated space-time yields [26, 27]. Additionally, this process mode allows for smaller cultivation vessels due to higher volumetric throughputs. Consequently, more comparable reactor sizes can be used in development and production scale, reducing the risk of scale-up effects. Further, operational costs decrease, and constant product yields can be achieved, neglecting time-dependent effects [20, 26–30]. Thus, smaller vessel sizes enable modular production facilities, reducing water and energy consumption and giving more adaptability and flexibility to current market situations [29, 30].

To further facilitate process optimization, process models can be developed, which are incorporating complex nonlinear interactions between process variables of interest in a condensed form [31]. Models can then be used to observe non-measured process variables in real-time by the means of soft-sensors [32] and to set up control strategies for the most crucial process variables in the process such as the product formation rate [33]. Using mathematical models, it is also possible to optimize process parameters by structurally analyzing the effects of the parameters and control inputs such as the feed rate on the quality attributes of the process [34]. The model-based concepts can lead to significantly reduced experimental efforts and enable a more knowledge-driven approach for process development.

To date, bacterial chemostat cultivations for recombinant protein production have been predominantly studied using the microbial host *Escherichia coli*. They proved to result in fluctuating productivity [35]. Subpopulations cause the fluctuating productivity, being investigated in detail in multiple publications [36–39]. However, a two-vessel continuous operation, kept subpopulations in a distinct manner, allowing for stable recombinant protein expression for over 200 h [40]. A high influence of the specific substrate uptake rate and the dilution rate on the productivity and stability of continuous cultivations was observed in *E. coli* continuous cultivation [40]. For other microbials, successful concepts of continuous cultivations were shown for *Komagataella phaffii* in chemostat and retentostat mode [41, 42]. Still other publications investigating an industrial *Saccharomyces cerevisiae* strain in long-term fermentation found metabolic and genomic shifts to cause reduced product formation [43].

In this presented study, the focus was to establish a stable continuous production process with a derepressible inductive system in order to generate a more economic and sustainable process alternative to fed-batch cultivations. Initially, we analyzed fed-batch cultivations of *B. licheniformis*, specifically examining the correlation between the specific substrate uptake rate (q_s_) and the specific protease productivity (q_p_) for a derepressible inductive system. Building on the knowledge gained from fed-batch processes, the study aimed to explore the advantages of continuous cultivation. Different dilution rates were tested in a non-dynamic manner and their effect on product formation was monitored for cultivation times exceeding 200 h. The overarching aim of this study was to determine whether a switch to continuous cultivation could confer advantages over fed-batch processes for the production of proteases by *B. licheniformis*. A hybrid model was established to describe the effects of dilution rate and generation time in regard to specific productivity and confirmed with reference runs. This research not only contributes to the existing knowledge of *Bacillus* fermentation but also paves the way for the potential transition to continuous cultivation methods for future industrial applications where efficiency and sustainability are increasingly paramount.

## Methods

### Strain and media

Clones of the recombinant protease-producing *B. licheniformis* were provided by BASF SE (Ludwigshafen am Rhein, Germany).

Two separate precultures were exercised: The first preculture consisted of the terrific broth (TB) medium as described by Habicher et al. [18]. The second preculture as well as the batch media used a defined medium described in WO/2020/169,564, supplemented with a trace element solution provided by BASF SE (Ludwigshafen am Rhein, Germany) [44]. The feed media for fed-batch cultivations consisted of the defined media consisting of 50% glucose w/w, with the detailed composition described elsewhere [44]. For chemostat cultivations, the batch medium was slightly adapted, however due to proprietary constraints, exact nutrient concentrations cannot be stated. Glucose concentration was set to 5% and maintained throughout all chemostat cultivations. Potential limitations have been controlled by timely measurements of the supernatant by inductively coupled plasma- optical emission spectrometry. Kanamycin was added to all cultivations to prevent plasmid loss at 20 µg/mL.

### Precultures

Two precultures have been performed as stated in Sect. 2.1. For preculture one, a sterile 250 mL Erlenmeyer shake flask with 10 mL TB medium was inoculated with 5% v/v of a frozen bacterial stock solution (stored at -80 °C) and incubated at 200 rpm and 30 °C for 15 h (Infors HR Multitron shaker, Infors, Bottmingen Switzerland). For preculture two, a 1 L Erlenmeyer flask with 50 mL defined medium was inoculated with 0.5% v/v of the first preculture. The culture was incubated at 300 rpm and 30 °C for at least 22 h (Infors HR Multitron shaker, Infors, Bottmingen Switzerland).

### Bioreactor cultivation

All cultivations were performed in a Minifors 2 bioreactor system (max. working volume: 1 L; Infors HT, Bottmingen, Switzerland). The offgas was analyzed in online mode using gas sensors – IR for CO_2_ and ZrO_2_ based for oxygen (BlueSens Gas analytics, Herten, Germany). Process control, exponential and constant feeding were executed using the process control system PIMS Lucullus (Securecell, Urdorf Switzerland). The pH was monitored using an EasyFerm PluspH-sensor (Hamilton, Reno, NV, United States) and was kept constant at 7 throughout all cultivations and controlled using solely base (12.5% NH_3_). Dissolved oxygen (dO_2_) was kept above 30% oxygen saturation by supplying a stirrer and aeration (pressurized air and pure oxygen) cascade. The dO_2_ was monitored using a Visiferm fluorescence dissolved oxygen electrode (Hamilton, Reno, NV, United States). Feed medium was added by using a preciflow pump (Lambda, Laboratory Instruments, Baar, Switzerland).

For the batch, 540 mL defined medium was sterilized at 121 °C for 20 min in the respective cultivation device. Prior to autoclavation, the pH probe was calibrated using a two-point calibration (pH 7 and pH 4). After sterilization, probes were connected and bioreactor settings (30 °C, 300 rpm, aeration of 0.8 vvm) were applied until inoculation. Following that, pump calibrations with the sterile feed medium were carried out prior to the feeding phase. Prior to inoculation, 2 mL antifoam were added as well as 9.6 mL feed solution (50% glucose w/w), in order to achieve a starting glucose substrate concentration of 8 g/kg. Moreover, the pH of the batch medium was set to pH 5.6 with 12.5% NH_3_ in a first step and then increased to the starting pH of 7.0 with 25% NaOH. Additionally, the dO_2_ probe was calibrated to 100%. Inoculation volume was performed with 10% of the batch starting volume. After the batch was over, indicated by a pH-shift, either the fed-batch process or the chemostat cultivation was started.

### Fed-batch

**Fig. 1:**
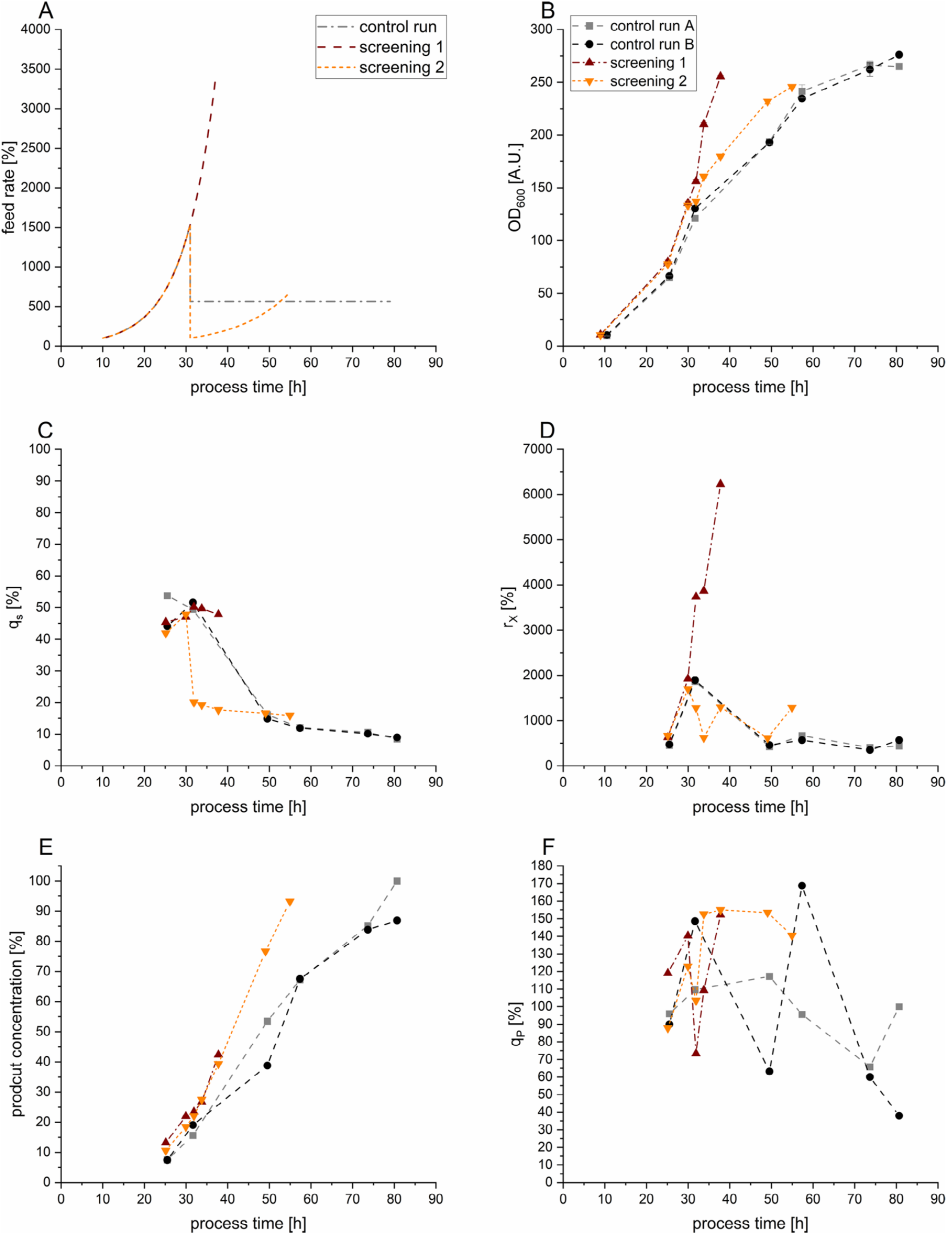
Results of different exercised fed-batch cultivations; the color code is: control run A , control run B , screening 1 , screening 2 . **A**: Applied feeding strategies. For the control runs an exponential feeding profile for 21 h was used, before a constant feed was applied. In screening 1 and 2 the effects of a different constant specific substrate uptake rate (q_s_) were examined (exponential feeding). **B**: Trend of the optical density measured at 600 nm (OD600) for the tested fed-batch cultivations. **C**: Specific substrate uptake rates (q_s_) normalized to the maximal substrate uptake rate of the cultivated strain. **D**: Biomass formation rate (r_x_). **E**: Trend of the obtained protease concentration over time, normalized to the titer achieved at the harvesting time point of control run A. **F**: Specific product formation rates (q_p_) normalized to the maximal q_p_ achieved in the control run A at the end of the fermentation

Fed-batch fermentations were performed with a 2-stage feed profile as described before [45] In the first phase, the feed medium was added exponentially for 21 h, subsequently either a constant feed rate (control runs) or an exponential ramp with a predefined specific growth rate (µ) was applied (Fig. 1A). Input values received from the pump calibration were put into the process control system PIMS Lucullus for the exponential ramp as well as the constant phase, respectively. Processes were aborted once an OD_600_ (absorption at 600 nm) of > 250 was reached.

### Chemostat

For the chemostat cultivation a dip-pipe was adjusted to the desired height, to maintain the operating volume, as described in literature [46]. After depletion of the supplemented carbon source, the dilution rate was set via the feed pump. Continuous settings were achieved by maintaining the bleed pump at a pump rate higher than the feeding pump. The continuous phase lasted for at least 200 h and was aborted then (Table 1).

**Table 1:** Overview of all performed chemostat cultivations. Dilution rate (D) is given in percent relative to the maximum specific growth rate µ [h^-1^]. The substrate specific uptake rate (q_s_) [g*g^-1^*h^-1^] are given in percent relative to the maximum q_s_

	training data set			validation data set		
	Chemostat 1	Chemostat 2	Chemostat 3	Chemostat 4	Chemostat 5	Chemostat 6
D [%]	24	39	72	19	49	57
q_s_ [%]	32	50	98	27	60	78

### Sample analysis

Samples were taken regularly throughout the cultivations, to monitor biomass growth and product formation. Sampling intervals were adapted to the corresponding cultivation types. The applied analytical procedures are described in detail below.

#### Biomass determination

Optical density was measured at an absorbance of 600 nm to monitor cell growth. The OD600 measurement was performed in triplicates with a Genesys 20 photometer (Thermo Scientific, Waltham, MA, USA), while samples were diluted with deionized water (dH2O) to be within the linear range of the photometer (0.2–0.8 AU).

#### Analysis of metabolites and substrate accumulation

To determine substrate and secondary metabolite concentrations, a Cedex Bio HT^®^ Analyzer (Roche CustomBiotech, Switzerland) was used. Supernatant samples, previously separated via centrifugation from the cell pellet, were analyzed with the Cedex Bio HT^®^. To be within the linear range of the assay, a predilution of the sample with ultrapure water (MQ) water was performed if required. Additionally, feed and cultivation samples were measured via HPLC (Thermo Scientific, Waltham, MA, United States) using a Aminex HPX-87 H Column (Bio-Rad, Hercules, CA, United States). Carbohydrates as well as organic acids were detected using a refractive index detector (Agilent Technologies, Santa Clara, CA, United States). The mobile phase consisted of 5 mM sulfuric acid operated at an isocratic elution profile at 0.5 mL/min. Respective standards were measured for quantification of each carbon source and expected secondary metabolite.

#### Protease assay

The enzymatic activity of the target protein in the supernatant of the collected samples was analyzed according to the assay described by Habicher et al. [47]. For all taken samples a serial dilution in a 96-well plate (Greiner 96 flat transparent plate, Greiner Bio-One, Kremsmünster, Austria) was prepared and subsequently measured in a plate reader (Tecan Spark 20 M, Tecan, Männedorf, Switzerland). For the assay a substrate stock of N-succinyl-alanine-alanine-proline-phenylalanine-*p*-nitroanilide (N-Suc-AAPF-pNA) at a concentration of 60 mg/mL in water-free dimethyl sulfoxide was prepared. For measurement, the stock was diluted 1 to 20 with 0.1 M Tris-HCL buffer containing 0.1% (w/v) Brij 35 at pH 8.6. Subsequently, 50 µL of the diluted samples were mixed with 100 µL of the substrate stock and the optical density was monitored at 30 °C and 405 nm for 15 min. Monitoring was performed with the SparkControlTM software and kinetics analyzed for slope and intercept. For evaluation, all values exceeding an absorption value of 2 were discarded and subsequent dilutions were performed if necessary. The protease activity was calculated using the change of absorption at 405 nm, the extinction coefficient of 8,900 M/cm, the used volumes and the pathlength of 0.47 cm. All samples were measured in triplicates and a protease standard was always used internally as a reference to ensure reproducibility in between measurements.

### Data normalization

Due to confidentiality and better comparability all data sets were normalized. Growth rates (µ [h^− 1^]), dilution rate (D [h^− 1^]) and the specific feeding rate (q_s_ [g*g^− 1^*h^− 1^]) are given in percentage, normalized to the maximal physiological values of the used strain. The biomass formation rate (r_x_ [g*h^− 1^]) is normalized to the average biomass formation in the batch. The product concentration is normalized to the highest achieved titer throughout all cultivations (control run A). Accordingly, all productivity results, like space-time yield (STY) and specific product formation rate (q_p_) are normalized to the same value (= end of cultivation value of control run A).

### Model identification and validation

The model identification was carried out using the programming language Julia version 1.9. The model describing the q_p_ is based on a fusion of kinetic terms taking into account the specific substrate uptake rate as well as the cumulative amount of metabolized substrate inspired by Kager et al. [33], which correlates with the cultivation time. The substrate uptake rate r_s_ can be calculated by balancing the incoming, outgoing and accumulating mass flows of substrate through Eq. 1.

1 $$\:{r}_{s}\left(t\right)={r}_{s,in}\left(t\right)-{r}_{s,out}\left(t\right)-{r}_{s,acc}\left(t\right)$$

r_s_ … substrate uptake rate [g*h^-1^]

r_s, in_ … substrate feeding rate [g*h^-1^]

r_s, out_ … substrate not taken up and transported out of the reactor [g*h^-1^]

r_s, acc_ … substrate accumulation rate [g*h^-1^]

The metabolic load (S_met_) can be obtained by integrating r_s_ over the course of the fermentation process (Eq. 2).

2 $$\:{S}_{met}\left(t\right)=\int\:{r}_{s}\left(t\right)dt$$

S_met_ … metabolic load [g]

r_s_ (t) … substrate uptake rate [g*h^-1^]

The values of r_s, in_, r_s, out_ and r_s, acc_ can in turn be estimated from online measured substrate feed rates, harvest rates and offline measured substrate concentrations. The specific substrate uptake rate q_s_ can then be calculated by dividing r_s_ by an estimated growth rate r_X_ using Eq. 3:

3 $$\:{q}_{s}\left(t\right)=\frac{{r}_{S}\left(t\right)}{X\left(t\right)}$$

q_s_ … specific substrate uptake rate [g*g^-1^*h^-1^]

r_s_ … substrate uptake rate [g*h^-1^]

X(t) … biomass amount [g]

and integration over time provides the cumulative specific amount of metabolized substrate (Eq. 4).

4 $$\:{q}_{s,cum}\left(t\right)=\int\:{q}_{s}\left(t\right)dt$$

q_s, cum_ … cumulative substrate uptake rate [g*g^-1^]

q_s_ … substrate uptake rate [g*g^-1^*h^-1^]

It is also possible to convert q_s, cum_ to the number of generations (n_gen_) by multiplying with the yield coefficient Y_X/S_

5 $$\:{n}_{gen}={q}_{s,cum}\left(t\right)\cdot\:\:{Y}_{X/S}$$

The substrate uptake rate q_s_ as well as the cumulative value q_s, cum_ were considered as two key descriptors for modeling the product formation rate q_p_ [40]. The model that was finally selected to calculate q_p_ from q_s_ and q_s, cum_ is shown below (Eqs. 5 and 6).

6 $$\:N\left({q}_{s}\right)=\frac{{p}_{1}}{{p}_{2}\sqrt{2\pi\:}}\cdot\:{e}^{-\frac{1}{2}({\frac{{q}_{s}-{p}_{3}}{{p}_{2}})}^{2}}$$

7 $$\:{q}_{p}\left({q}_{s},{q}_{s,cum}\right)=N\left({q}_{s}\right)\cdot\:{e}^{-{p}_{4}\cdot\:{q}_{s,cum}}$$

π … Pi, 3.14

N … distribution of q_P_ in the q_S_ dimension [g*g^-1^*h^-1^]

q_s_ … substrate uptake rate [g*g^-1^*h^-1^]

q_P_ … specific product formation rate [g*g^-1^*h^-1^]

q_s, cum_ … cumulative substrate uptake rate [g*g^-1^]

This model puts the product formation rate in correlation with the metabolic activity (described by the substrate uptake rate) as well as the number of generations (described by q_S, cum_). The parameters p1 to p4 were identified using an unconstrained Nelder-Mead-Simplex optimization procedure based on the training data set (Table 2) [48]. More specifically chemostat processes 1,2 and 3 have been used for parameter identification (model training), whereas the remaining processes were used to validate the model. The identified parameter values are listed in the table below.

**Table 2:** Identified parameter values for the established model

Parameter	Identified value
p1	5.45
p2	0.136
p3	0.331
p4	0.0138

## Results

### Studying the effects of growth rates and substrate uptake rates on product formation

#### Fed-batch cultivation

As catabolism controls target protein expression, the q_s_ is linked to biomass growth and induction, therefore being pivotal in adjusting biomass formation and protein expression levels. Initially, two control experiments at industrial standard process settings comprising of a dual feeding phase were performed, to determine reproducibility between fed-batch cultivations. Feeding segments were encompassing an exponential phase to increase biomass levels before a subsequent constant feed at a lower feeding rate was exercised to induce protein expression (Fig. 1A).

Two further screening runs were conducted employing a constant q_s_ to investigate the effect of alternating feeding rates on protein expression. In the screening 1 cultivation, a q_s_ of approximately 50% resulted in accelerated biomass growth, reaching the targeted optical density (OD600 of > 250) within 38 h. The control runs A and B took 80 h, whereas the screening 2 run needed 55 h to reach the target OD (Fig. 1B). Despite this expedited growth, a comparable product titer was achieved after approximately 35 h (Fig. 1E). The increased feed rate conducted in screening run 1, resulted in the highest biomass formation rate (Fig. 1D), but contrary in a lower productivity compared to screening run 2. Both processes were conducted at exponential feeding rates and led to a notable increase in q_p_, which exceed the end-of-fermentation values of control run A, by reaching 119% and 141%, respectively.

In the control runs, the q_s_ showed the anticipated decrease starting at 30 h, in line with the constant feeding rate applied. Intriguingly, even with an exponential feeding profile, a declining q_s_ was noted, indicating variations in biomass yield over time. While the q_p_ displayed fluctuations, a noticeable declining trend over cultivation time could be observed even though q_s_ was maintained constant (Fig. 1F).

#### Chemostat cultivations

**Fig. 2:**
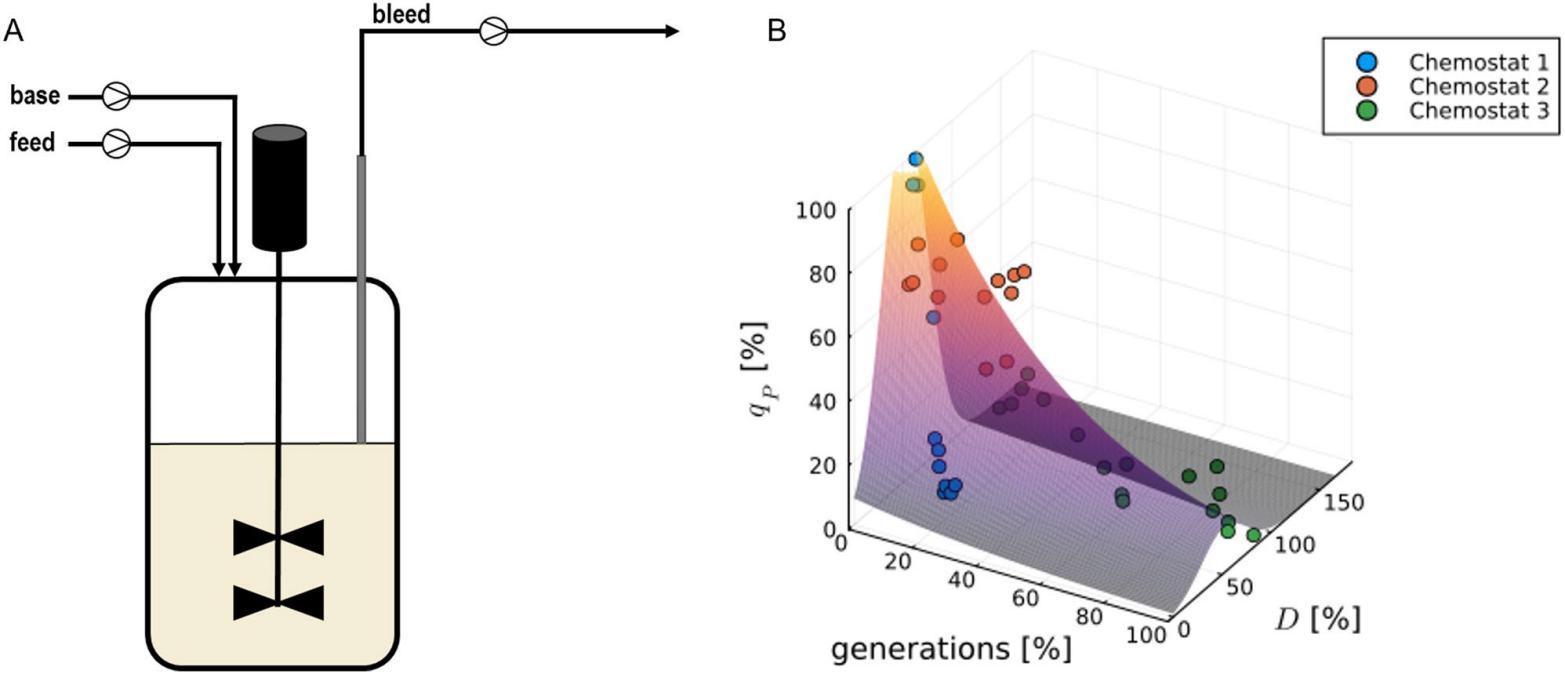
**A**: Schematic sketch of the chemostat cultivation set-up. A dip-pipe is adjusted to the required volume height. A bleed pump operated at higher pump speed than the feed pump ensures for a constant dilution rate (D). **B**: Model identification of the performed chemostat cultivations with a D of 1: 24%, 2: 39%, 3: 72% of the maximum specific growth rate (µ [h^-1^])

Chemostat cultivations were systematically studied varying the dilution rate (D) only, using a fixed substrate concentration, to examine its effects on biomass growth and product expression (Fig. 2A). As a result of the fed-batch screening, we proceeded with a series of bioreactor experiments at low (D = 24%), medium (D = 39%), and high (D = 72%) dilution rates (Table 1). Cultivations were specifically assessed in regard to their long-term productivity investigating the relationship between D and q_p_. The data acquired from these experiments were instrumental in developing a model that delineates the dynamics between q_s_, q_s, cum_ and q_p_ (Fig. 2B).

The initial chemostat cultivations revealed that an intermediate dilution rate of approximately 40% resulted in a q_s_ of 50%, which was found conducive to product formation. Nonetheless, when the D and q_s_ were operated below 25%, there was a noticeable decrease in productivity. At a dilution rate of D = 72% of the maximum uptake rate protein formation was significantly hindered. Biomass formation remained consistent across the cultivation process. In conclusion, there was a pronounced effect of the duration of cultivation on productivity, as the induction continued, a gradual decline in protein concentration was noted. As can be seen in Fig. 2B, the model formulation produces a normally distributed behavior in the dimension of D (N(q_s_), Eq. 6) and exponentially decreasing q_p_ in the direction of increasing generations, corresponding to q_s, cum_ (Eq. 7). This was found to be the most accurate model formulation given amount and uncertainty of the available data while also trying to use a minimal amount of model parameters in order to ensure parameter identifiability and avoid overfitting.

### Model validation and detailed chemostat screening

**Fig. 3:**
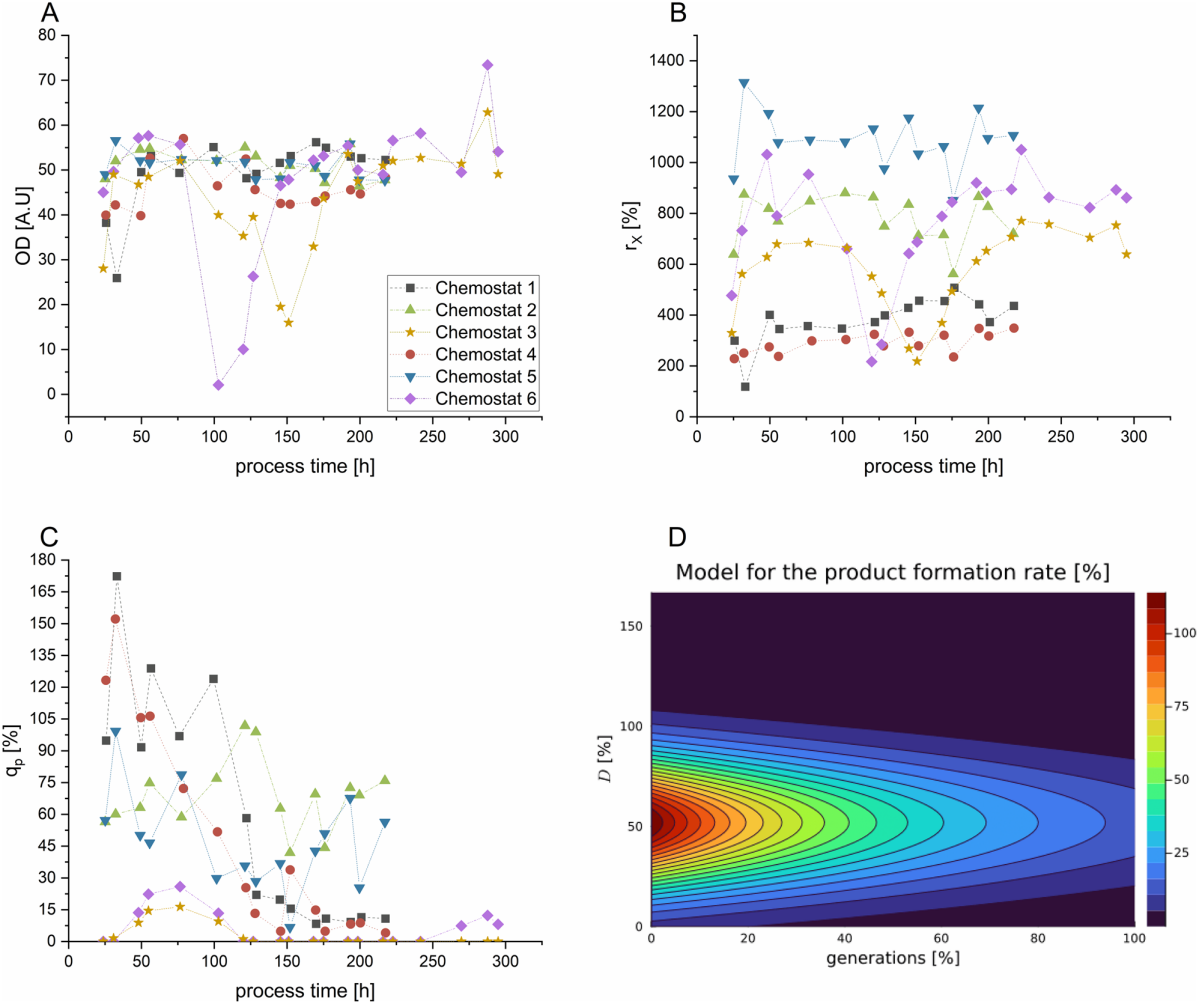
Overview of all performed chemostat cultivations. **A**: Trend of the optical density measured at 600 nm (OD600). **B**: Biomass formation rate (r_x_). **C**: Space-time yield (STY) in % relatively to the STY in [g*L^-1^g*h^-1^]at the end of the fed-batch control run A. **D**: Contour Plot predicting the product formation rate in % relatively to the achieved product formation rate (q_p_[g*g^-1^g*h^-1^]) at the end of the fed-batch control run A. Chemostat 1 (D = 24%), Chemostat 2 (D = 39%), Chemostat 3 (D = 72%), Chemostat 4 (D = 19%), Chemostat 5 (D = 49%), Chemostat 6 (D = 57%)

The commencement of our chemostat cultivations was aimed at establishing a robust model to depict product formation, alongside a detailed analysis of how process parameters influence the key product-related metric (q_p_). Prediction and modeling of STY were excluded for the modelling part as the main focus was set on generating mechanistic knowledge. To validate the formulated model and the inferred parameter influences, three subsequent validation runs were carried out. These were performed at dilution rates of 19%, 49%, and 57%, with corresponding q_s_ values of 25%, 60%, and 78%, to cover a broad spectrum of operational conditions (Fig. 3).

Overall, at lower dilution rates (D = 19% and 24%), cell growth appears to stabilize post initial adaptation, as reflected by the relatively steady OD values (Fig. 3A). This stability is corroborated by the biomass formation rates (r_x_) detailed in Fig. 3B. Conversely, there is a noted decline in the q_p_ starting after approximately 50 to 70 h (Fig. 3C). When operated at dilution rates of 39% and 49%, r_x_ values resulted in higher to previous cultivations, whilst maintaining the most consistent q_p_, indicating an optimal balance between nutrient supply, cellular growth and protein expression. At the highest dilution rates tested, over-foaming caused an inadvertent rise in the dilution rate. This caused a washout, as indicated by decreasing OD_600_ values, therefore these processes were operated for an extended cultivation time. Despite the process deviations, q_s_ values reaching 98% (D = 72%, Chemostat 3) and 78% (D = 57%, Chemostat 6), repressed the promoter, resulting in a decreased product formation as seen in Fig. 3C. Hence results show that the model could be validated within the intended design space by applying the model to the three cultivations, that were not used for parameter identification. The differences of model accuracy with respect to the training, validation and total dataset is displayed in Table 3.

**Table 3:** Summary of the obtained normalized root-mean-square error (NRMSE). The normalization procedure was carried out through division by the difference between the maximum and the minimum product formation rates

Data provided	NRMSE [%]
Training dataset (Chemostat 1,2,3)	14.2
Validation dataset (Chemostat 4,5,6)	18.1
Total dataset	15.1

## Discussion

In contemporary biomanufacturing, fed-batch cultivations play a pivotal role. However, in order to achieve sustainable bioprocesses, it requires continuous small scale operations, to reduce water consumption, energy consumption and greenhouse gas emissions [49]. A shift to continuous cultivation, can improve equipment utilization and offers benefits such as smaller equipment, easier scalability, lower costs, better product quality and long-term productivity [23, 24].

Conducted fed-batch cultivations showed a strong effect of q_s_ in regard to r_x_ and q_p_: Fig. 1 indicated exponential feeding at low µ to boost product formation, still specific productivity decreased over time. We attribute the cellular machinery to be encountering metabolic burden associated with sustained protease expression, leading to a decline in q_p_ over time [50]. Owing to the derepressed expression system, 10% of the product titer was already measurable at the end of the batch phase, already resulting in a certain product burden for host cells prior to the induction phase [11]. Additionally, phenotypic and/or genetic heterogeneities could contribute to variations in protease expression levels, impacting overall productivity, depending on the number of generations achieved in cultivation [15, 51].

Comparing processes operated with an exponential feeding to processes operated with a constant feeding rate revealed that the exponential feeding strategy resulted in a noteworthy 30% increase of the STY. We attribute this effect to the higher amount of energy being applied, whilst still maintaining derepressed conditions, in comparison to the constant-fed reference processes. Due to the depressed promoter system, a critical threshold of q_s_ is given. Once the promotor experiences repression, it is delineating the balance required for optimal expression and biomass growth. However, studies addressing carbon derepressed induction systems with *B. licheniformis* are scarce in literature.

Still, productivity was always observed in all fed-batch cultivations. As we investigated for specific feeding rates up to 50% of the maximum specific substrate uptake rate (q_s, max_), results indicated promotor derepression to be possible up until 50% of µ _max_ (Screening 1, Table 4). These results imply a substantive connection between q_s_ and q_p_, and were thus utilized for further processes in the chemostat cultivations.

To test for the effect of D on q_p_ in chemostat cultivations, we operated three processes at D = 24%, 40% and at 72%, respectively. The process operated at a dilution rate of approximately 40% resulted in a q_s_ of 50% (Chemostat 2), remaining within the derepressed system as shown in the conducted fed-batch cultivations. Chemostat cultivation 1 and 3, showed an adaptation phase with an initial rising q_p_ before decreasing over the number of generations. Unlike observations reported in previous studies with *E. coli*, where productivity could diminish to zero, the process operated at D = 23% maintained at a low, yet steady level of productivity over the duration of the cultivation assessed [21, 35]. We attribute this to the rise of non-producing subpopulations, as stated for multiple microbial processes [26, 37]. Although plasmid copy numbers were not monitored, selective pressure was consistently applied throughout all cultivations, minimizing plasmid loss [15]. The process operated at D = 72%, showed initially a lower productivity compared to chemostat 1, however q_p_ decreased and maintained at 0 after approximately 120 h of process time. We attribute the lower q_p_ to result from weakened induction as a result of the derepressed system in comparison to the other two conducted chemostat experiments. All processes ran unobtrusive except for an instance of overfoaming in chemostat 3 after 150 h and chemostat 6 after 100 h, which led to an unintentional increase in the dilution rate and subsequent washout. However, the biomass concentration recuperated after 30 h and remained stable for the remainder of the process. Metabolic fluctuations in continuous cultures have been determined for *B. subtilis*. Cellular distribution of the energetic precursors could potentially explain the fluctuations in host cell productivity [52].

With these datasets an initial mechanistic description of the chemostat cultivations was performed [53]. The model uncertainty was the lowest in the training dataset (14.2%), normalized through division of the difference between the maximum and the minimum product formation rates, being plausible as the model was identified using this very dataset [54]. To validate the dataset, three more chemostat cultivations were conducted:

The process operated at D = 19% showed similar characteristics to the process conducted at D = 24%. Both processes were conducted at a similar feeding rate compared to the fed-batch screening 2 run. Whereas screening run 2 led to the highest q_p_, the performed chemostat cultivations resulted in a low average q_p_, due to the decrease of productivity over cultivation time. Both chemostat cultivations operated at a low D (chemostat 1 and 4) peaked in initial productivity, before declining over process time. We attribute this phenomenon again to occurring subpopulations, trying to escape the high pressure of induction as reported for multiple microbials [36, 37, 55]. Still productivity maintained at a q_p_ of 10% +/- 1% for chemostat 1 and at 8% +/- 4% for chemostat 4 in average after 170 h. Intermediate dilution rates (chemostat 2 and 5) however showed a fluctuating, however steady effect in regard to q_p_. This could have been caused by lower induction of the promotor keeping the production system in place, whilst maintaining physiological properties of the cells. Interestingly these results are also contrary to the fed-batch cultivations: initial productivity in chemostat cultivations was lower for cultivations at intermediate dilution rates in comparison to the peaking productivity observed at processes operated at a low dilution rate. For fed-batch cultivations in screening 1 and 2, no differences were observed in regard to q_p_. We hypothesize that the effect of strong derepression might be thus generation dependent, indicating a declining q_p_ starting from approximately 30–35 generations as observed in Fig. 3C&D.

High dilution rates (chemostat 3 and 6) resulted in a rapidly decreasing productivity, showing similar kinetics compared to *E. coli* chemostat cultivations. Moreover, with q_s_ values reaching 98% (D = 72%, Chemostat 3) and 78% (D = 57%, chemostat 6), the promoter should be theoretically repressed in a time-dependent behavior, resulting in an absence of product formation as seen in Fig. 3C.

Even though higher volumetric fluxes increase the theoretical mass throughput in continuous cultivations [21], in the context of a derepressed induction system, the q_s_ must be carefully chosen to initiate product formation without compromising biomass growth. Notably, at a dilution rate of 49% with a q_s_ of 60% (Chemostat 5), product formation was still initiated, whereas at a slightly elevated dilution rate of 57% and a q_s_ of 78% (Chemostat 6), time-dependent repression occurred. This suggests, that the threshold q_s_ of avoiding repression is within the boundaries of these operated conditions. The validation dataset (chemostat 4–6) showed just a slightly higher uncertainty of NRMSE = 18.1%, which was expected as these data were used to validate the initial training data set. Putting all data together, the NRMSE was found to be 15.1%, which is only 1% higher than the error received for the initial training dataset. Therefore, the validation was considered successful and the model can be used to predict productivity in chemostat cultivation when varying the dilution rate and the generation time [56]. This, in turn, enables a model-based process design by testing certain processing conditions using the model instead of real lab experiments. Different, dynamic, feeding strategies could be tested on the model regarding the resulting product formation rate and a mathematical optimization procedure could then be performed in order to find the optimal feeding profile to maximize the product formation rate. The results lead to a bell shaped- productivity curve, as low dilution rates result in potential subpopulation fluctuation and high dilution rates are assumed to cause promotor repression. Hence results align with literature where protease production via *B. lichenoformis* has been monitored for 20–30 h in chemostat cultivations [57]. Thereby low dilution rates resulted in the highest cell specific productivity, however we attribute this effect only to be monitored due to the reduced time-span of cultivation.

The contour plot (Fig. 3D) underscores once more, a pronounced dependency of the productivity on the duration of cultivation and the dilution rate —a finding that is in accordance with results from other microbial hosts in continuous cultivation previously reported [26, 40]. Chemostat 2, operated at a dilution rate of 39% with a q_s_ of 50%, demonstrated the highest average STY and q_p_, of the conditions tested (Table 4). However, Chemostat 1 and Chemostat 3, with q_s_ values of 32% and 98% respectively, show diminished STYs, with Chemostat 6 dropping to 0%, indicating a complete promoter repression. In contrast, Chemostat 2, operated a moderate q_s_ of 50%, demonstrated a balanced outcome with a q_p_ of 69% and a STY of 46%.

**Table 4:** Comparison of fed-batch and chemostat cultivations. µ/D: values in percent relative to the maximum specific growth rate µ [h^-1^]. For control run A and B a range is given due to a constant feeding profile. q_s_ [g*g^-1^*h^-1^]: Values in percent relative to the maximum specific substrate uptake rate. For control run A and B at the beginning of the constant feeding profile is given. Specific productivity (q_p_) [g*g^-1^*h^-1^]: Values are normalized to the achieved specific product formation rate in control run A at the end of the fermentation. The depicted values are the average for the whole process as soon as constant process conditions had been achieved. Space-time yield (STY) [g*L^-1^*h^-1^]: Values are the overall STY for the total process duration for fed-batch and the average in the constant phase for chemostats. All values are normalized to the achieved STY in control run A

	Fed-batch cultivations				Continuous cultivations					
	control run A	control run B	Screening 1	Screening 2	Chemostat 1	Chemostat 2	Chemostat 3	Chemostat 4	Chemostat 5	Chemostat 6
µ/D [%]	16 – 8	15 - 9	48	18	24	39	72	19	49	57
Process time for calculation [h]	50 - 81	50 - 81	25 - 38	25 - 55	33 - 218	32 - 217	199 - 295	56 - 218	49 - 217	141 - 295
q_s_ [%]	12*	12*	48 +/- 1	18 +/- 2	32 +/- 7	50 +/- 4	98 +/- 5	25 +/- 2	60 +/-3	78 +/- 6
q_p_ [%]	95 +/- 21	83 +/- 59	119 +/- 31	141 +/- 22	56 +/- 56	69 +/- 17	0	29 +/- 32	43 +/- 19	2.5 +/- 5
STY [%]	94	84	80	131	30 +/- 22	46 +/- 11	0	21 +/- 16	33 +/- 10	2 +/- 3

Overall, the aim of the study was to establish chemostat cultivations as a viable alternative to fed-batch processes. This however, was challenged by the complexity of balancing growth rates, and productivity. When comparing fed-batch cultivations to chemostat cultivations (Table 4), the STY of conducted chemostat cultivations is always below any conducted fed-batch cultivation. In addition to that, qp also fluctuated for chemostat 2 & 5, despite achieving the lowest deviation on average. Hence results indicated, chemostat cultivations need to be operated (i) in a way that higher volumetric product can be achieved and (ii) in a more stable manner in order to allow for a steady downstream process and make full use of continuous cultivations [29, 58]. While exponential feeding in screenings showed promise in enhancing q_p_ and STY, the chemostat runs revealed different trends with a highly fluctuating productivity. Hence, our results indicate that for derepressible systems, variations in q_s_ might have different effects in the chosen cultivation type. Contrary to prior investigations involving *E. coli* and a T7-based induction system, it is apparent that fed-batch cultivations are unsuitable for screening optimal growth and production conditions to be applied in continuous cultivations [40, 59]. For derepressible systems, techniques are necessitating careful adjustment to harness the impact of population dynamics and induction strength in continuous cultivation. Genetic mutations, altered transcript expression potentially elucidating cellular stress responses were not part of this study, however could be investigated in follow-up studies, to further elucidate the strain’s physiology. We suggest to investigate the potential of cascaded continuous cultivations in future follow-up studies, to spatially separate biomass growth and protein expression [40]. Alternatively, the use of cell retention systems [58, 60] might be potentially feasible to improve product formation for this derepressed induction system in a continuous manner.

## Conclusion

Our results indicate that in a derepressed induction system, a narrow operational range that supports either biomass growth or induction is essential for effective continuous cultivation. A clear dependence on generation time was observed, likely due to the formation of subpopulations during prolonged induction periods. For chemostat cultivations, a dilution rate between 24% and 50%, with a specific substrate uptake rate (q_s_) exceeding 30%, was necessary to achieve stable productivity for recombinant protease production. This, however is highly different to results obtained from fed-batch cultivations, where low growth rates were facilitating productivity. Hence, the results state that the influence of the feeding rate on specific productivity varies significantly between fed-batch and chemostat cultivations. This difference suggests that fed-batch screenings alone are insufficient to develop a continuous process for a derepressed promoter system in *B. licheniformis*.

Hence, the experimentally developed dynamic process model could be of use to streamline future designs that aim to develop continuous cultivation processes for different target products. Still, results of this study indicate fed-batch cultivations to substantially achieve higher STYs than chemostat processes, making them the preferred method for producing recombinant proteases with *B. licheniformis*.”

## Data Availability

All data is provided within the manuscript and due to confidentiality and better comparability all data sets were normalized.
